# Similar Long-Term Outcomes in Children Presenting With Abscess vs Phlegmon at Diagnosis of Crohn Disease

**DOI:** 10.1093/crocol/otaa060

**Published:** 2020-07-20

**Authors:** Andrew W Fondell, Maua H Mosha, Ross M Maltz, Brendan M Boyle, Barbara Joanna Niklinska-Schirtz, Subra Kugathasan, Meghan E Gibson, Jason M Shapiro, Sarah M Rosenheck, Annette L Langseder, Mary C Kennedy, Joel R Rosh, Jeffrey S Hyams

**Affiliations:** 1 Division of Digestive Diseases, Hepatology, and Nutrition, Connecticut Children’s, Hartford, Connecticut, USA; 2 Division of Pediatric Gastroenterology, Hepatology and Nutrition, Nationwide Children’s Hospital, Columbus, Ohio, USA; 3 The Center of Microbial Pathogenesis, Abigail Wexner Research Institute, Nationwide Children’s Hospital, Columbus, Ohio, USA; 4 Division of Pediatric Gastroenterology, Children’s Healthcare of Atlanta, Atlanta, Georgia, USA; 5 Division of Pediatric Gastroenterology, Nutrition, and Liver Diseases, Hasbro Children’s Hospital, Providence, Rhode Island, USA; 6 Department of Pediatric Gastroenterology, Goryeb Children’s Hospital-Atlantic Health, Morristown, New Jersey, USA; 7 Department of Clinical Development and Research Affairs, Goryeb Children’s Hospital-Atlantic Health, Morristown, New Jersey, USA

**Keywords:** Crohn disease, abscess, children, inflammatory bowel disease, phlegmon

## Abstract

**Background:**

Limited data are available for long-term outcomes of pediatric patients with abdominal abscess or phlegmon at diagnosis of Crohn disease.

**Methods:**

We performed a retrospective chart review of such children over a recent 6-year period at 5 pediatric inflammatory bowel diseases.

**Results:**

Fifty-two patients (mean age 15.9 ± 1.8 years) were reviewed. Thirty-six had an abscess and 27 (75%) required resectional therapy compared to 16 with phlegmon which 10 (63%) requiring surgery. Overall (37/52) 71% had surgery which was performed within 6 months in 32 (86%).

**Conclusions:**

A similar high surgical rate exists whether pediatric patients with Crohn disease present with abscess or phlegmon.

## INTRODUCTION

Inflammatory mass with or without accompanying abscess formation can be seen at initial presentation of Crohn disease (CD) in children.^1^ Diagnostic evaluation with cross-sectional imaging is often helpful to delineate the type of inflammatory mass.^2–4^ Whether differentiation between phlegmon and abscess has important implications for treatment decisions is unclear as both have historically been considered to represent penetrating (B3) CD. Classically, a phlegmon has been defined as an acute suppurative tissue inflammation that may undergo liquefaction necrosis eventually resulting in an abscess.^5^ The interobserver agreement between radiologists in the diagnosis of phlegmon vs abscess has been shown to be excellent across multiple imaging modalities including computerized tomography (CT), magnetic resonance imaging (MRI), and contrast-enhanced ultrasound.^2^ On imaging, a phlegmon appears as a hypoechoic mass with poor margination within the inflamed echogenic perienteric fat while abscesses are present as hypoechoic fluid collections with an irregular wall.^2^

Initial management often involves intravenous antibiotics and when needed abscess drainage. Both medical and surgical management have been successfully employed for these patients. We were interested in examining whether there were differences in long-term outcomes of pediatric patients presenting with intra-abdominal abscess vs phlegmon. Accordingly, we reviewed a multicenter experience with these types of patients at 5 large pediatric inflammatory bowel disease (IBD) centers focused on short- and long-term outcomes, acute medical vs surgical management, and long-term medical therapies.

## MATERIALS AND METHODS

### Study Design and Patient Selection

This is a multicenter retrospective medical chart review of patients 4–18 years of age diagnosed with CD who on initial presentation with cross-sectional imaging were found to have an inflammatory mass consisting of an abscess or phlegmon. Phlegmon was defined as an ill-defined mass-like inflammatory process of soft tissue, while abscess was defined as a fluid collection with rim enhancement with or without internal gas.^6^ Patients were seen between January 1, 2013 and April 30, 2019 with a minimum of 6 months of follow-up. Participating centers reviewed institutional databases to identify appropriate patients. Inpatient and outpatient records were reviewed and de-identified data were extracted into a central REDCap database.

Collected data included age, sex, clinical disease severity at diagnosis by Pediatric Crohn’s Disease Activity Index (PCDAI),^7^ disease location based on the Paris classification system,^8^ laboratory data (white blood cell count, hemoglobin, platelet count, erythrocyte sedimentation rate, C-reactive protein, and serum albumin), and body mass index (BMI). Relevant imaging study reports including modality were reviewed, as were duration of hospitalization, antibiotic usage and duration, need for percutaneous drainage (PD), maintenance medical therapy, and any surgical intervention. Patients were followed from 6 months up to 5 years as complete data were available.

### Outcomes

We defined the primary outcome as the need for resectional surgery within 1 year of presentation. Secondary outcome included the use of anti-tumor necrosis factor (TNF) therapy designated either prior to surgery, following surgery, or use with no subsequent need for surgery.

### Statistical Analysis

Descriptive statistics were used to evaluate patient characteristics and outcomes of disease reported in proportions, means ± SD, or medians with interquartile ranges (IQRs). Kaplan–Meier was used to describe survival rate of being free from surgery. Independent *t* test for comparisons of means across 2 groups were utilized. All statistical analyses were performed using SPSS software ver. 26.0 (IBM, Armonk, NY) and Microsoft Excel 2013 for Windows (Microsoft, Redmond, WA).

### Ethical Considerations

This study was reviewed and approved by the Institutional Review Board of each participating center.

## RESULTS

### Patient Characteristics

A total of 52 patients are included in this study. Patients were divided into 2 groups based on the presence of phlegmon or abscess on cross-sectional imaging at the time of diagnosis. Clinical, demographic, and laboratory characteristics at diagnosis are shown for both groups in Table 1. There were 36 (69%) patients with an abscess and 16 (31%) with phlegmon. The mean age was 15.9 ± 1.8 years and 30 (58%) were female. Duration of follow-up was 5 years for 8 patients (15%), 1 year for 41 (79%), and 6 months for 3 (6%). Symptom duration of more than 1 month prior to presentation was seen in 34 (65%), 13 (25%) from 1 week to 1 month, and 5 (10%) with less than 1 week of symptoms.

**TABLE 1.** Baseline Characteristics of Study Population at Diagnosis

Variable	All Patients (N = 52)	Abscess (n = 36)	Phlegmon (n = 16)	*P*
Age (y) (±SD)	15.9 (1.8)	15.9 (1.94)	16.0 (1.4)	
Female	30 (58%)	18 (50%)	12 (75%)	0.13
Stricturing and penetrating disease behavior (B2B3)	11 (21%)	8 (22%)	3 (19%)	1.00
Abdominal pain duration prior to diagnosis				0.24
<1 week	5 (10%)	3 (8%)	2 (12%)	
1 week to 1 month	13 (25%)	7 (20%)	6 (38%)	
>1 month	34 (65%)	26 (72%)	8 (50%)	
BMI* (kg/m^2^) (±SD)	19.9 (5.4)	18.8 (4.1)	22.6 (7.0)	0.02
PCDAI^†^ (±SD)	42.9 (12.5)	43.2 (14.0)	42.5 (9.64)	0.86
Laboratory values (±SD)				
White blood cell count (T/µL)	13.0 (5.87)	13.1 (6.1)	12.8 (5.5)	0.86
Hemoglobin (g/dL)	10.5 (1.64)	10.6 (1.7)	10.6 (1.5)	0.94
Platelets (T/µL)	477 (172)	483 (179)	466 (159)	0.75
C-reactive protein (mg/dL)	7.91 (5.87)	7.35 (5.91)	9.16 (6.13)	0.39
Erythroycte sedimentation rate (mm/h)	45.9 (30.6)	44.4 (29.6)	49.6 (33.5)	0.58
Albumin (g/dL)	3.29 (0.62)	3.20 (0.66)	3.49 (0.46)	0.12
Ileal inflammation on imaging^‡^ (cm) (±SD)	20.4 (10.2)	22.7 (12.0)	17.5 (6.3)	0.41
IBD^§^-related surgery	37 (71%)	27 (75%)	10 (63%)	0.51
Timing to surgery from diagnosis	n = 37	n = 27	n = 10	
≤6 months	32 (87%)	24 (89%)	8 (80%)	
6 months to 1 year	2 (5%)	2 (7%)	0	
≥1 year	3 (8%)	1 (4%)	2 (20%)	

Data expressed as mean (±SD) or n (%).

*Body mass index.

^†^Pediatric Crohn’s Disease Activity Index.

^‡^Imaging includes ultrasound, CT, and MRI.

^§^Inflammatory bowel disease.

**FIGURE 1.**
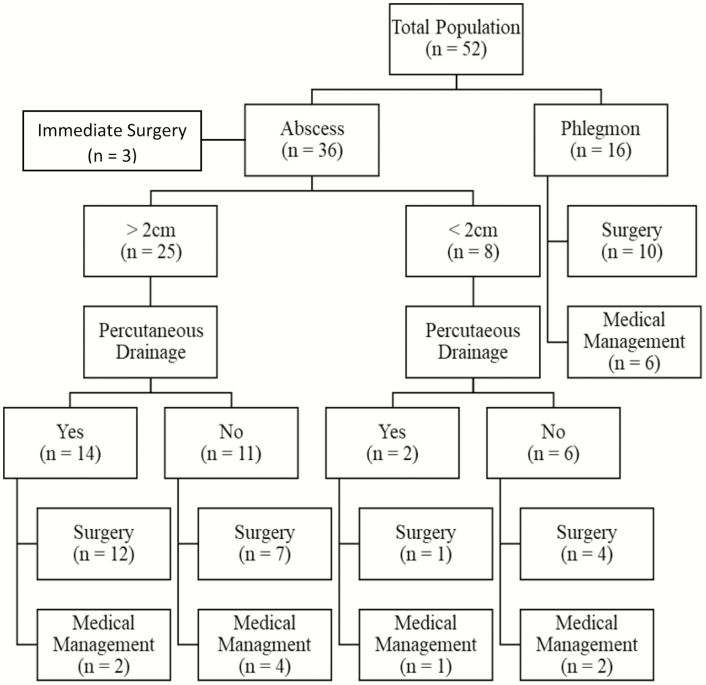
Study flowchart.

**FIGURE 2.**
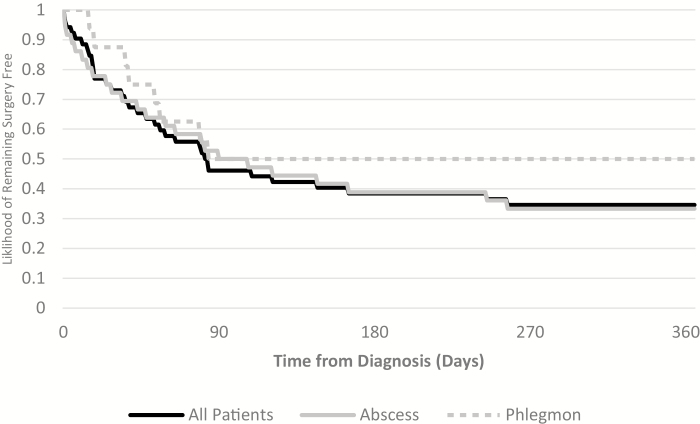
Survival curve for patients remaining surgery free in first year of diagnosis.

For the entire study population, 37 (71%) patients underwent surgery during follow-up; 27 (75%) of those with abscess and 10 (63%) of those with phlegmon (*P* = 0.51). Short- and long-term management of our study population is shown in Figure 1. The likelihood of remaining surgery free for the 2 study groups in the first year following diagnosis is shown in Figure 2.

### Patients With Phlegmon

Thirteen patients had a right lower quadrant phlegmon and 3 had a central abdominal phlegmon at diagnosis. There were 6 patients (37%) with phlegmon that received medical management alone. Mean age was 16.4 ± 1.6 years, 5 were female, mean BMI 27.8 ± 7.8 kg/m^2^, and mean PCDAI 45.5 ± 9.3. Cross-sectional imaging showed none had stricturing disease and mean ileal inflammation of 18 ± 2.5 cm. Three of these patients were discharged home from diagnostic hospitalization on IV antibiotic therapy administered for 4–6 weeks, 2 were on enteral antibiotics for 4–6 weeks, and 1 on enteral antibiotics for less than 4 weeks. One patient was discharged home on exclusive enteral nutrition (EEN). Five patients were started on infliximab with a mean initiation time of 50 ± 37 days from CD diagnosis and 1 on thiopurine monotherapy.

There were 10 (63%) with phlegmon that underwent surgery during study time frame. Mean age 15.8 ± 1.3 years, 7 were female, mean BMI 19.5 ± 4.1 kg/m^2^, and mean PCDAI 45.5 ± 9.3. Mean ileal inflammation was 17 ± 8.5 cm and 3 had stricturing and penetrating disease (B2B3) on cross-sectional imaging. Four patients were discharged home on IV antibiotics; 6 patients had a less than 4 week course of antibiotics and 3 had 4–6 weeks of therapy. One patient was placed on EEN.

All patients underwent ileocecectomy with 1 also having segmental small bowel resection. Surgery was performed at a median of 54 (IQR 14–82) days with 8 (80%) undergoing procedure within 6 months of diagnosis. One patient encountered a postsurgical complication which was a wound infection. No patient had an ostomy placed and none required a second operation. Of the 4 patients started on a biologic prior to surgery, it was initiated at a range of 2–20 days following CD diagnosis and patients underwent surgery at 35, 83, 612, and 758 days from diagnosis. Six patients underwent surgery prior to initiating maintenance IBD therapy with mean timing to surgery of 42 ± 24.5 days from diagnosis, with a range of 14–78 days. Five of these patients were then started on biologic therapy, mean time from surgery to initial dose was 41 ± 5.1 days. One patient was started on thiopurine monotherapy.

### Patients With Abscess

There were 36 patients with an abscess with 8 measuring less than 2 cm. There were 3 patients with a small abscess that avoided surgery during study time frame. Mean age 16.9 ± 1.3 years, all were males, mean BMI 15.4 ± 2.6 kg/m^2^, and mean PCDAI 49 ± 11.8. On cross-sectional imaging, none had stricturing disease, and mean ileal inflammation was 16.9 ± 10 cm. One patient underwent PD. Two patients were discharged home on IV antibiotics with duration of 4–6 weeks and 1 on oral antibiotics for less than 4 weeks. All patients were started on infliximab at mean of 36.7 ± 12 days from CD diagnosis. No patient was started on EEN.

There were 28 patients with an abscess greater than 2 cm at diagnosis with 6 patients avoiding surgery during study time frame. Mean age of 14.9 ± 1.8 years, 4 females, mean BMI 19.1 ± 4.2 kg/m^2^, and mean PCDAI 28 ± 25. One patient had stricturing and penetrating disease. Two patients underwent PD. One patient was discharged home on IV antibiotics for 4–6 weeks, 2 with enteral antibiotics for 4–6 weeks, 1 for longer than 6 weeks, and 2 for less than 4 weeks. No patients were on EEN. Five patients were started on infliximab and 1 on adalimumab with median time to initiation of 5 days (IQR 1–11) from CD diagnosis.

Twenty-seven (75%) of the 36 patients with an abscess went to surgery. Mean age 16 ± 2 years, 14 females, mean BMI 19 ± 4.1, and PCDAI 44.4 ± 11.9. Mean length of ileal inflammation was 23.9 ± 12.4 cm and there were 7 patients with stricturing and penetrating disease. All patients that underwent surgery had an ileocecectomy, with 1 patient also having a segmental small bowel resection and 2 others had resection of the sigmoid as well. The median time to surgery for patients with an abscess was 42 days (IQR 14.5–98.5) with 24 (89%) within 6 months. One patient had an ileostomy placed that was reversed after 4 months. Four patients (15%) encountered complications after surgery with 1 having an anastomotic leak and bowel obstruction, 1 patient had an anastomotic leak, 1 had a wound infection, and 1 had a pleural effusion. Two of these 4 patients required a second surgery related to their complications.

Eleven of these patients were started on anti-TNF therapy and then went to surgery with mean timing of 148.7 ± 131.3 days from diagnosis, with a range of 16–463 days. There were 16 patients that went to surgery prior to starting an anti-TNF with 3 patients going to surgery within 24 hours of diagnosis and the remaining 13 patients at a mean of 37.8 ± 35.5 days from diagnosis, with a range of 4–120 days.

## DISCUSSION

Prior studies have shown a cumulative incidence of surgery for pediatric patients newly diagnosed with CD to be at 6% at 1 year and 17% by 5 years^9^; however, these studies generally do not distinguish patients presenting with B3 disease from others developing B3 during disease course. Our multicenter data suggest that the presence of abscess or phlegmon at presentation is associated with a surgical rate over 70% and is similar whether the patient has either a phlegmon or abscess at diagnosis. This number is similar to 1 prior adult study revealing surgical rates in patients with CD and phlegmon at 58%.^10^ Though not distinguishing intra-abdominal abscess at presentation or later in the disease course, a pediatric study of CD demonstrated a 64% rate of surgical intervention.^11^ For our study population, 16 patients presented with a phlegmon for which 10 (63%) required surgery and 36 patients presented with an abscess with 27 (75%) undergoing surgery (*P* = 0.51). Our study uniquely included only those pediatric patients presenting with penetrating (B3) disease at CD diagnosis. These patients required a high rate of surgery despite management strategies such as early introduction of anti-TNF, PD, and prolonged antibiotics to treat inflammatory masses.

Prior literature has shown that early initiation of anti-TNF therapy in pediatric patients with nonstricturing, nonpenetrating CD were less likely to develop penetrating complications.^12^ There are little data on whether early initiation of anti-TNF therapy in pediatric patients presenting with penetrating disease can delay or potentially avoid surgery. As seen in our patient group, there did not appear to be a decrease in surgical rates with earlier initiation; however, providing anti-TNF prior to surgery appeared to extend timing from diagnosis to ileocecectomy. The mean length of time from CD diagnosis to surgery was 208 ± 58.6 days for patients initiated on anti-TNF prior to surgery vs 33.9 ± 6.9 days for those that went to surgery prior to anti-TNF. Potentially this extended period may allow for additional bowel healing and less extensive resection. There were no identifiable demographic or clinical findings to identify which patients that were started on an anti-TNF would avoid surgery and which would not. Potential reasons for patients to require surgery appeared to be multifactorial including failure to resolve signs and symptoms despite biologic or steroid therapy and also extent of ileal inflammation.

Percutaneous abscess drainage is often used in patients with pelvic or abdominal abscesses to allay infection, improve efficacy of antibiotics, potentially decrease need for surgery, or mitigate complexity of resection.^13^,^14^ Our multicenter study showed that despite PD in addition to IV antibiotics, 14 (82%) of the 17 patients underwent surgery during study time frame. Prior studies have shown the need for IBD-related surgery at 60%–90% for pediatric patients despite undergoing PD.^2,7,8^ These prior studies included a combination of known and recently diagnosed CD compared to our study of only pediatric patients presenting with an abscess which further shows the difficulties in treating these patients that show signs of perforation at time of diagnosis.

Baseline demographics at initial diagnosis between abscess and phlegmon groups revealed they were relatively similar with only mean BMI between the 2 groups showing a statistical difference. Patients with an abscess had a mean BMI of 18.8 ± 4.1 kg/m^2^ and those with a phlegmon 22.6 ± 7.0 kg/m^2^ (*P* = 0.02). There were no significant differences in PCDAI, length of ileal inflammation on imaging, inflammatory markers, albumin, or results from complete blood count tests.

We sought to identify features at diagnosis that might predict avoidance of surgery. Not surprisingly, we found that the presence of an associated stricture was a poor prognostic sign. Based upon imaging studies at the time of diagnosis, there were 11 patients diagnosed as B2B3 with 10 (91%) undergoing surgery. There were 3 patients with a phlegmon and 8 with an abscess; of which 1 patient with an abscess avoided surgery. Compared to the 41 patients with B3 disease alone, 26 (63%) underwent surgery vs 91% of B2B3 revealing presence of narrowing at diagnosis had a higher surgical rate.

Prior literature has shown a wide variation in diagnostic and therapeutic interventions in penetrating CD^15^; however, since the release of the NASPGHAN clinical report on internal penetrating CD in 2013^5^ there has been improvement in standardization of care between centers. As observed between the 5 involved pediatric IBD centers, there was only modest variability in treatment patterns. There was divergence on length of antibiotic timing and use of home parenteral antibiotic therapy but choices in antibiotics were similar across the study centers.

## LIMITATIONS

Our data collection was retrospective and original data entry from the patient medical records may have missing or incomplete components that could affect the quality and completeness. Interpretation of cross-sectional imaging and techniques used (ultrasound, CT, and MRI) were not standardized. However, the study group is the largest collection of pediatric CD patients presenting with an abscess or phlegmon at time of diagnosis with long-term follow-up that we could identify. We were able to observe treatment trends and outcomes within these patients; however, due to the modest sample size we may have been unable to recognize significant factors that lead to or potentially avoid need for surgical intervention.

## CONCLUSIONS

The rate of surgical intervention for pediatric patients presenting with phlegmon or abscess at time of CD diagnosis is similarly high. Though anti-TNF agents were used in some patients in conjunction with abscess drainage when appropriate and intravenous antibiotics, the majority of these patients still required resectional surgery. Prospective controlled trials of optimized medical therapy will be needed to determine better staging criteria for patients who have a higher likelihood of avoiding surgery.

## Data Availability

Data available on request due to privacy/ethical restrictions.

## Notes

Conflicts of interest: These authors disclose the following: SK has been a consultant for Janssen and UCB. JRR has been a consultant for AbbVie, Bristol Meyers Squibb, Celgene, Janssen, Lilly, Luitpold, and Pfizer, and received grant funding from Janssen and AbbVie. JSH has served on an advisory board for Janssen and is acting as a consultant for AbbVie, Takeda, Lilly, Boehringer-Ingelheim, Allergan, Pfizer, Receptos, and AstraZeneca. The remainder of the authors have no financial disclosures.
